# An Overview of Long Non-Coding (lnc)RNAs in Neuroblastoma

**DOI:** 10.3390/ijms22084234

**Published:** 2021-04-19

**Authors:** Francesca Baldini, Matilde Calderoni, Laura Vergani, Paola Modesto, Tullio Florio, Aldo Pagano

**Affiliations:** 1 Department of Experimental Medicine, University of Genova, 16132 Genova, Italy; baldinifrancesca92@gmail.com (F.B.); calderoni.matilde@gmail.com (M.C.); 2 Department of Earth, Environment and Life Sciences DISTAV, University of Genova, 16132 Genova, Italy; laura.vergani@unige.it; 3 National Reference Center for Veterinary and Comparative Oncology-Veterinary Medical Research Institute for Piemonte, Liguria and Valle d’Aosta, 10154 Torino, Italy; Paola.Modesto@izsto.it; 4 IRCCS Ospedale Policlinico San Martino, 16132 Genova, Italy; tullio.florio@unige.it; 5 Department of Internal Medicine (DIMI), University of Genova, 16132 Genova, Italy

**Keywords:** neuroblastoma, lncRNAs, cancer

## Abstract

Neuroblastoma (NB) is a heterogeneous developmental tumor occurring in childhood, which arises from the embryonic sympathoadrenal cells of the neural crest. Although the recent progress that has been done on this tumor, the mechanisms involved in NB are still partially unknown. Despite some genetic aberrations having been identified, the sporadic cases represent the majority. Due to its wide heterogeneity in clinical behavior and etiology, NB represents a challenge in terms of prevention and treatment. Since a definitive therapy is lacking so far, there is an urgent necessity to unveil the molecular mechanisms behind NB onset and progression to develop new therapeutic approaches. Long non-coding RNAs (lncRNAs) are a group of RNAs longer than 200 nucleotides. Whether lncRNAs are destined to become a protein or not, they exert multiple biological functions such as regulating gene expression and functions. In recent decades, different research has highlighted the possible role of lncRNAs in the pathogenesis of many diseases, including cancer. Moreover, lncRNAs may represent potential markers or targets for diagnosis and treatment of diseases. This mini-review aimed to briefly summarize the most recent findings on the involvement of some lncRNAs in NB disease by focusing on their mechanisms of action and possible role in unveiling NB onset and progression.

## 1. Introduction

Neuroblastoma (NB) is a malignant pediatric solid tumor that originates during embryonic or early post-natal life from the sympathetic cells derived from the neural crest [1]. NB is the most common extracranial tumor occurring in childhood [2]; over 30% of cases are diagnosed in infants and the remaining, mostly, under five years of age [3]. NB is characterized by remarkable heterogeneity, in terms of phenotype and localization. It can arise in several areas of the body: most of the cases develop in the abdominal region, especially from the adrenal glands, but can also develop in the chest, neck or along the spinal cord [2]. Due to its complexity and heterogeneity, NB can show extremely different clinical behavior.

Several genomic alterations have been identified in NBs leading to different patterns of clinical behavior. Historically, NB subtypes were classified into different stages based on multiple factors such as genetic alterations, age of patient, presence of metastasis, etc. Stages 1, 2 and 4S described tumors with little or no risk and favorable prognosis. Instead, stages 3 and 4, known as high-risk NBs (HR-NBs), are characterized by aggressiveness, low response to therapy and poor prognosis [4,5].

Recently, a new NB classification was proposed by Ackermann et al. [6] who found that alterations in telomere maintenance mechanisms as well as in RAS or p53 pathways better discriminate between high-risk or low-risk NBs than the previous classification. Telomeres are responsible for genomic integrity in normal cells, and telomere length and telomerase activity are crucial for cancer initiation and tumor survival. In particular, Ackerman et al. showed that survival rates were lowest for NB patients whose tumors harbored telomere maintenance mechanisms in combination with *RAS* and/or *p53* mutations. On the other hand, low-risk NBs did not show telomere maintenance mechanisms, in the absence of which the possible mutations in *RAS* or *p53* genes seem to not affect patient outcome [6].

A clear example of neuroblastoma heterogeneity is the different clinical outcomes, ranging from spontaneous regression or differentiation into a benign ganglioneuroma to unremitting and aggressive progression despite multimodal therapy [7]. The mechanisms underlying the spontaneous regression are currently unknown, and a better understanding of this process may help to identify new therapies.

The real etiology of this tumor is still unknown, but the sporadic form represents most of the cases, whereas only 1–2% of affected children present a genetic autosomal dominant inheritance pattern [8]. NB can show a broad range of chromosomal abnormalities, but the most common genetic alteration is the amplification of the oncogene *MYCN*, which is observed in 20–25% of cases and in 50% of high-risk tumors [9]. Another genetic aberration, found in 9% of primary NB, is the activation of the anaplastic lymphoma kinase (*ALK*) gene [3]. All these mutations are associated with poor clinical outcome, but, in a few cases, they represent possible therapeutic targets.

Important molecules used for NB therapy act on the membrane targets GD2 and B7-H3. GD2 is a disialoganglioside expressed on the membrane of numerous cancer cells, such as brain tumors, retinoblastoma, osteosarcoma and NB [10]. Anti-GD2 monoclonal antibodies are currently used in therapy to improve standard treatments for HR-NBs, but further studies are necessary to confirm the effectiveness of this immunotherapy and to optimize it [11]. However, in 12% of patients with bone marrow relapse, NB cells lose GD2 expression, thus rendering the use of this treatment impossible [12]; for this reason, Dondero et al. [13] developed a multiparametric flow cytometry to observe GD2 surface expression, suggesting B7-H3 targeting therapy for those patients in which GD2 is missed. B7-H3 is a transmembrane glycoprotein overexpressed in NB cells (particularly in bone marrow aspirates) [14], as well as in melanomas, gliomas and breast and pancreatic cancers [15]. B7-H3 is a member of the B7 family that may down-regulate natural killer (NK) cell cytotoxicity through binding to NK receptors, leading to the activation of inhibitory signals. Recently, a murine IgG1 antibody against B7-H3 (omburtamab) was tested for NB therapy, showing significant effectiveness in NB patients with central nervous system involvement [15]. A phase II/III study is still ongoing [16].

Despite the therapeutic approach advancement in recent years, NB still represents 15% of all pediatric cancer deaths [17], and a comprehensive and detailed view of molecular and genetic mechanisms that bring to NB development is not available yet. For this reason, NB represents a significant unmet medical need and a challenge in terms of prevention and treatment, highlighting the importance of exploring new molecular pharmacological targets, such as non-coding RNA, especially for HR-NBs.

**Figure 1:**
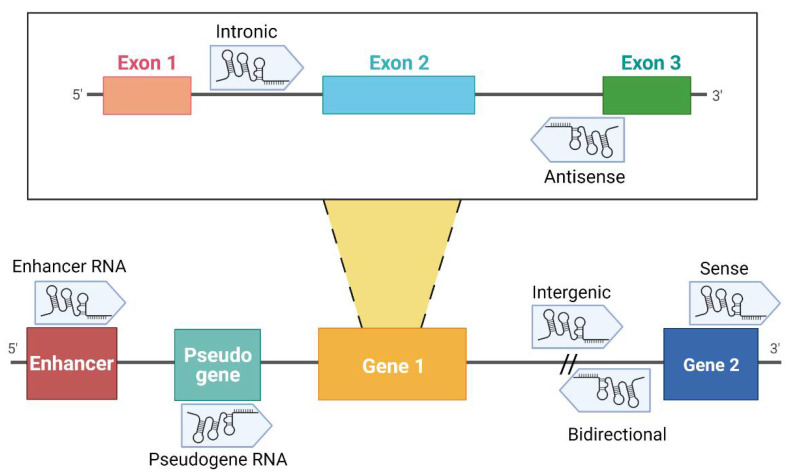
Classification of lncRNAs according to their genome localization. Intronic: the sequence originates from an intron region of a protein-coding gene [19]. Antisense: transcribed from the antisense strand of a gene sequence, originating from an exon or intron region [20]. Enhancer RNA (eRNA): RNA transcribed from transcriptional enhancer. eRNAs could present polyadenylation and 5′ cap. Generally, they are unstable with a short half-life [21,22,23]. Pseudogene RNA: the transcripts originated from pseudogene and could be short or long ncRNAs [24,25]. Intergenic: the localization is in an intergenic region, precisely more than 1 kb away from closest genes [20,22,26]. Bidirectional: the sequence is mainly located on the opposite strand with respect to a gene, of which transcription starts less than 1000 bp away [26,27,28]. Sense: this kind of lncRNA is transcribed from the sense strand and contains exons of protein-coding genes [29]; some are variants of mRNAs, while others do not contain a functional open reading frame [28]. Created with BioRender.com (accessed on 9 April 2021).

Over the past decades, it has become evident that the non-coding portion of the genome plays a fundamental role in many diseases and in cancer in particular. Non-coding RNAs (ncRNAs) are functional transcripts that regulate gene expression at a transcriptional and post-transcriptional level. ncRNAs are classified as housekeeping RNAs (such as rRNA, tRNA) and regulatory RNAs such as microRNA (miRNA), piwi-interacting RNA (piRNA) and long non-coding RNA, which differ in terms of length [18]. While lncRNAs are longer than 200 nucleotides, miRNAs are approximately 22 nucleotides in length. Recently, circular RNAs (circRNAs) have been also identified as gene regulators; their circular structure is due to the linkage between 3′ and 5′ ends of a single-stranded RNA molecule [18]. A division of lncRNAs is reported in Figure 1.

Despite the biological functions of many ncRNAs still being largely unknown, lncRNAs have been shown to be potentially involved in multiple cancer types [23,30,31], including NB [8,32,33]. Over the last year, high-throughput approaches became a powerful tool to identify a pool of lncRNAs that are differentially expressed by NB cells.

Here, we summarized the most recent findings regarding lncRNAs that have been proposed to be associated with NB, focusing on their effects and mechanisms of action within tumor cells. Our study focused on the more promising NB-associated lncRNAs that could represent reliable biomarkers for identifying the onset and/or progression of high-risk or malignant NB, thus representing a possible tool for future works in NB.

## 2. Main lncRNAs Down-Regulated in Neuroblastoma

### 2.1. FOXD3-AS1

Zhao et al. [34] analyzed public microarray datasets of NB cases and identified five lncRNAs consistently associated with progression and aggressiveness of NB and patients’ death. Among them, lncRNA forkhead box D3 antisense RNA 1 (FOXD3-AS1) resulted to be the most interesting. FOXD3-AS1 is an independent prognostic marker for positive outcome in NBs: FOXD3-AS1 is down-regulated in NB tissues at advanced stages or with poor outcome, compared with normal dorsal ganglia. The authors confirmed this observation using an in vitro approach; FOXD3-AS1 was expressed at a low level in NB cell lines and in correspondence with *MYCN* amplification. Stable transfection of FOXD3-AS1 led to a reduction in NB cell viability and invasiveness and promotion of neuronal differentiation. Using an RNA immunoprecipitation (RIP) approach, they demonstrated that FOXD3-AS1 directly interacted with PARP1 protein, a member of the PARP family, which plays crucial functions in DNA repair, genomic integrity and gene regulation [34]. *PARP1* overexpression in NB cells increased their invasion and proliferation rates, and both effects were counteracted by stable transfection with FOXD3-AS1. Co-immunoprecipitation (co-IP) was then performed to identify which PARP1-interacting protein was the target of FOXD3-AS1, and the results show that FOXD3-AS1 represses the PARP1-mediated PARylation of CCCTC-binding factor (*CTCF*) which plays an oncogenic role in NB. Indeed, CTCF binds the promoter of genes involved in cancer such as *p53*, *c-Myc* and *retinoblastoma* and regulates their expression across epigenetic mechanisms. Finally, Zhao et al. showed that the treatment with FOXD3-AS1 construct or with siRNAs against *PARP1* or *CTCF* reduces tumor growth and extends mice xenografts’ survival, confirming the causal relationship between these molecules.

Guan et al. [35] demonstrated that FOXD3-AS1, acting as an oncogenic regulator, could be a potential diagnostic or prognostic biomarker in breast cancer. Its high expression in breast cancer tumors is correlated with cell proliferation, migration and invasion.

Cervical cancer (CC) with high expression of FOXD3-AS1 was associated with lymphatic invasion, distant metastasis and poor overall survival. In CC, FOXD3-AS1 modulates the progression of the tumor through the expression of miR-296-5p targeting *HMGA1* [36] or targeting and negatively regulating miR-128-3p, which indirectly up-regulated *LIMK1* expression [37].

According to a study by Wang (2020), in osteosarcoma, FOXD3-AS1 expression is higher than in normal tissue, and its lack inhibits cell migration, invasion and epithelial-mesenchymal transition via the absence of FOXD3-AS1 sponging activity of miR-296-5p [38].

In nasopharyngeal carcinoma, FOXD3-AS1 influences the tumor progression and metastasis presence by negative modulation of the miR-185-3p expression [39].

### 2.2. NBAT1 and CASC15

Although NB pathogenesis is still largely unknown, cell differentiation is a key point during this process, and an improper differentiation may lead to tumor formation [40]. Genome-wide association studies (GWASs) led scientists to uncover many NB risk loci strongly associated with NB development and aggressiveness [41,42,43,44]. The 6p22 region of the genome has been highlighted as an NB hotspot since it harbors a cluster of SNPs associated with an increased risk of NB [45]. It is interesting to highlight that the genes located in this locus, *CASC15* and *NBAT1*, promote differentiation through the regulation of cancer-associated genes [46]. Indeed, downregulation of 6p22 lncRNAs in NB cell lines leads to perturbation of neuronal differentiation [46].

NBAT-1, the lncRNA neuroblastoma-associated transcript-1, has been identified as an independent prognostic marker for the clinical outcome in patients with NB [46,47], non-small cell lung cancer (NSCLC) [48] and in oesophageal cancer [49], and studies also proposed its association with hepatocellular carcinoma (HCC) [50], renal carcinoma [51,52], lung [53] and breast cancer [54], tumorigenesis, proliferation, migration and invasion [47]. Loss of NBAT-1 has been observed in NB cells, and in particular, a significantly lower expression has been highlighted in HR-NB. Downregulation of NBAT-1 resulted to be differentially modulated in NB subtypes by both genetic and epigenetic factors. High-risk-associated SNP (rs6939340) on 6p22, in the intron 2 of the *NBAT-1* gene, parallels the loss of expression of the gene [45,47]. Furthermore, in high-risk patients, *NBAT-1* promoter was found to be hypermethylated, thus suggesting that epigenetic regulation is also involved in the regulation of the expression of this lncRNA in NB cells [47]. Remarkably, NBAT-1 exerts tumor suppressor activity through the regulation of several genes which are known to be involved in the development of a wide range of cancers, including *SOX9*, *OSMR* and *VCAN*. In particular, NBAT-1 interacts with *EZH2*, thus repressing the gene expression via epigenetic mechanisms [47,55]. Furthermore, NBAT-1 plays also a role in neuronal differentiation induced by retinoic acid (RA). In vitro, NB cells exposed to RA showed a significant increase in NBAT-1 expression [55]. Pandey et al. [47,55] clarified the role of this lncRNA in this process, suggesting an involvement of the NRSF/REST pathway during NBAT-1-induced proper neuronal differentiation. NB cells with low NBAT-1 expression show a consequent upregulation of the NRSF/REST pathway resulting in reduced expression of key neuron-specific genes. Moreover, NBAT-1 regulates p53 subcellular localization, promoting p53 accumulation in the cytoplasm when it is down-regulated, leading to resistance to genotoxic drugs in NB cells [56]. Therefore, in the light of all these observations, NBAT-1 and its downstream effectors, involved in tumor suppressor and neuron differentiation activities, can be considered as potential therapeutic targets.

As previously described, 6p22 locus harbors also the *CASC15* gene which encodes for a lncRNA. A low expression of a CASC15 variant, CASC15-003, directly correlates with poor prognosis in NB patients [46]. Although *CASC15* involvement in NB onset and progression has still to be fully clarified, much research has shown that this lncRNA is closely related to many other kinds of tumors, acting as a potential driving gene with an oxymoronic expression modulation. CASC15 has been observed to be abnormally high-expressed in some tumors, including CC, breast cancer [57], gastric cancer [58], leukemia [59] and melanoma [60], to cite a few. Conversely, CASC15 is down-regulated in ovarian cancer [61], glioma [62] and NB, as detailed before.

Since both *CASC15* and *NBAT1* genes are located in the same locus, and both CASC15-003 isoform and NBAT1 are correlated with NB, their possible functional cooperation has been examined. Remarkably, the overexpression of *NBAT1* in *CASC15-003* knock-down cells rescued the differentiated phenotype, and vice versa, without affecting the expression of the other lncRNA [46]. This observation suggests that these two lncRNAs are fundamental and cooperate as complements for proper neuronal differentiation. While NBAT-1 mechanisms of action have been more studied and elucidated, the functional role of CASC15 and its isoforms needs to be explored.

### 2.3. DLX6-AS1

Public microarray datasets represent a powerful tool and a mine of information for scientists. Among all the lncRNAs detected by microarrays analyses, DLX6-AS1 has been observed to be up-regulated in both NB tissues and cell lines [63]. Deepening this observation, high expression of DLX6-AS1 was positively correlated with poor differentiation and advanced NB stage and, therefore, with a poor outcome [63,64]. Moreover, the upregulation of DLX6-AS1 has been correlated to the proliferation, migration and invasion in lung adenocarcinoma [65], gastric cancer [66], colorectal cancer (CRC) [67], breast cancer [68], bladder cancer [69], ovarian cancer [70] and osteosarcoma [71].

Knockdown of DLX6-AS1 induces neuronal differentiation and cell apoptosis, slows invasion in vitro and diminishes tumor growth in vivo [63]. These observations indicate that this lncRNA might play an oncogenic role in NB onset and progression.

### 2.4. NDM29

Neuroblastoma Differentiation Marker 29 (NDM29) is a non-coding RNA transcribed by RNA polymerase III, that can be classified as a long non-coding RNA [72]. It is an Alu-like RNA, and it maps in the first intron of the *ASCL3* gene, located in the 11p15.3 region, which is frequently deleted in NB [73].

NDM29 over-expression in the *MYCN*-amplified NB cell line (SKNBE2) leads to differentiation and loss of malignity [74]. NDM29 synthesis is significantly increased in differentiating cells, causing a slowdown of cell cycle progression and a reduction of the proliferation capacity [74,75].

The gradually increased NDM29 expression has been observed to be directly correlated with a progressive differentiation toward a neuronal phenotype. Cells develop neuron-like features, such as morphology and a complex network of neuritic processes [76]. On the other hand, as a counterpart of cell differentiation, malignant cells reduce their proliferation rate while showing excitatory properties [74,77].

The multiclonal cell model obtained by the stable and gradual over-expression of this non-coding RNA well recapitulates NB cell differentiation stages and represents a powerful tool to investigate potential novel pharmacological targets [74,78].

What is even more interesting, in light of a possible therapeutic approach, is that stable overexpression of NDM29 also makes cells more vulnerable to the action of antitumor drugs, such as cisplatin and doxorubicin. In fact, NDM29 down-regulates multi-drug reactivity 1 (*MDR1*) expression, a cell surface pump involved in detoxification and whose activity is involved in chemoresistance [78].

Moreover, by employing the NDM29-base model, Garbati et al. were able to identify a panel of genes progressively up-regulated from the neuron-like cells to the malignant stage and vice versa. Among the genes associated with the malignant stage, minichromosome maintenance complex 2 (*MCM2*) and carbonic anhydrase 9 (*CA9*) show a positive correlation with tumor growth, and these genes are potential targets for therapeutic strategy. Recent in vivo studies showed that the use of MCM2/CA9 inhibitors, such as ciprofloxacin and acetazolamide, respectively, in combination with cisplatin, improve mice overall survival and reduce tumor nodule growth [78].

Altogether, these observations suggest that this lncRNA can promote neuronal differentiation on NB cells and can be considered as a promising target for NB therapy and for unveiling NB onset and progression.

## 3. Main lncRNAs Up-Regulated in Neuroblastoma and Correlated with MYCN Amplification

### 3.1. LncNB1

In 2019, Liu et al. [79] identified an lncRNA over-expressed in *MYCN*-amplified NB cell lines. This transcript was named “lncRNA highly expressed in neuroblastoma 1” (lncNB1). LncNB1 expression is elevated only in a proportion of NB tumors and is moderate in skin melanoma but lower or absent in other cancer tissues.

An analysis of how lncNB1 affects gene expression regulation demonstrated that *DEPDC1B* gene expression and E2F1 protein expression are up-regulated by this transcript [79]. DEPDC1B induces ERK phosphorylation which, in turn, enhances N-Myc protein stability. In particular, lncNB1 leads to an increase of E2F1 protein expression by binding to the ribosomal protein RPL35, and this mechanism induces *DEPDC1B* gene transcription. This regulation is fundamental for NB cell proliferation and survival because silencing one of these components leads to a reduced percentage of NB cells in the S phase of the cell cycle and to an increase in apoptotic cells. Finally, lncNB1 knockdown considerably improved mice overall survival. Indeed, high levels of lncNB1 expression, together with *DEPDC1B*, RPL35 and E2F1 predict poor prognosis in NB patients [79].

### 3.2. SNHG1

Small nucleolar RNA host gene 1 (SNHG1) is involved in several human cancers’ regulation, such as CRC, HCC, lung, prostate and oesophageal cancers, glioma and NB [80]. In particular, it is associated with various carcinogenesis processes: cell proliferation, invasion and metastasis. However, its role in carcinogenesis is different according to the various types of tumors: in CRC, it is considered an oncogene and is involved in Wnt/β-catenin pathway regulation [81]; in HCC, it inhibits *p53* expression and p53-target genes [82]; and in NSCLC, it regulates miR-101-3p/SOX9/Wnt/β-catenin axis [83]. Furthermore, Chen et al. described a different pathway in the SHSY5Y NB cell line in order to better understand the role of α-synuclein pathology in Parkinson’s disease. Briefly, they proposed that SNHG1 targets miR-15b-5p activating seven in absentia homolog 1 gene and promoting α-synuclein aggregation [84].

Zhang et al. explored the mechanism regulated by SNHG1 in NB, demonstrating that it interacts with miR-338-3p, whose role in targeting proto-oncogenes in different cancers has been reported [85]. In NB cells, miR338-3p regulates *PLK4*, probably inhibiting its expression. On the other hand, SNHG1 expression induces downregulation of miR338-3p, leading to *PLK4* overexpression, promoting proliferation, migration and invasion [85].

Sahu et al. identified SNHG1 as a lncRNA up-regulated in *MYCN*-amplified NB [86]. In particular, they analyzed the expression of an RNA-sequencing dataset consisting of 493 patients, identifying different transcripts whose expression correlates with *MYCN* status. They validated these results by performing RT-qPCR in NB cell lines *MYCN*-amplified and *MYCN*-non-amplified. SNHG1 was found to be highly positively correlated with *MYCN*-amplified, together with *TAF1D*, a coding gene, both in cell-line and high-risk NB patients. Finally, they proposed SNHG1 expression levels as a prognostic biomarker in predicting the clinical outcome of NB patients.

### 3.3. SNHG16

Overexpression of “small nucleolar RNA host gene 16” (SNHG16), also called ncRAN (non-coding RNA expressed in aggressive neuroblastoma) [87], is correlated with bad prognosis in different types of cancer, including bladder cancer [88], breast cancer [89], glioma [90], HCC [91], osteosarcoma [92] and pancreatic cancer [93], being involved in the regulation of apoptosis, migration and proliferation of cancer cells [87].

Various studies demonstrated that SNHG16 can act as a competitive endogenous RNA (ceRNA), that is, a mechanism through which ncRNA can competitively bind different miRNAs, regulating the expression of miRNA target genes. However, the pathways involved are different according to the tumor examined. Furthermore, SNHG16 might have a transcriptional role through epigenetic modification: for example, in CC, SNHG16 can recruit SPI1, a transcription factor for *PARP9*, leading to the development of cancer [94]. Despite numerous studies that involved SNGH16 in cancer, further information is necessary to understand this lncRNA mechanism of activity and its potential diagnostic use [87].

Recently, different research groups studied the role of SNHG16 in NB. Here, we report some examples. In 2019, by analyzing GEO datasets, Yu et al. [95] demonstrated that SNHG16 is associated with poor prognosis in NB. They proposed that this lncRNA regulates cell cycle progression, cell proliferation and migration. In 2020, Zhaoying et al. [96] studied the pathway involved in SNHG16 activity. In particular, they focused their attention on cisplatin-resistant NB cells, finding out that SNHG16 was overexpressed in both tissue and cells resistant to this drug. Then, they explored the molecular mechanism by which SNHG16 targets miR-338-3p and their role in NB carcinogenesis: they demonstrated that PLK4, an important regulator in the duplication of the centriole, was positively regulated by SNHG16 by sponging miR-338-3p. In turn, this axis can induce the activation of PI3K/AKT, among other pathways, contributing to the resistant phenotype. Bao et al. identified another miRNA regulated by SNHG16, miR-128-3p, which serves as a tumor suppressor in NB. SNHG16 negatively modulates miR-128-3p, preventing its interaction with its targeted gene *HOXA7*, a sequence-specific transcription factor involved in many human cancers [97]. Finally, Wen et al. suggested that SNHG16 expression level was connected with the INSS stage and *MYCN* status. These results demonstrate that SNHG16 up-regulated *ATG5* via sponging miR-542-3p, increasing cell proliferation rate, migration and autophagy in NB cells [98].

Altogether, these papers described different pathways in which SNHG16 is involved as a sponge for other miRNAs, but no one proposed a therapeutic approach using the newly acquired knowledge.

## 4. LncRNA Associated with NB Regression

In a recent study [99], some lncRNAs associated with spontaneous regression of NB were identified. The novelty of this work was the attempt to identify survival-related candidates and to choose the transcripts that could be prognostic for spontaneous regression of NB, independently from *MYCN* amplification, age and INSS stage for survival of NB patients. They collected data from two independent databases, performing analyses on samples from patients with stage 4 and death outcome and from patients with stage 4S and survival outcome. They identified 20 lncRNAs that are differently expressed between the two cohorts: four were up-regulated in stage 4 NB samples and defined as “bad survival lncRNAs”, while sixteen were up-regulated in 4S NB samples and called “good survival lncRNAs”. The most interesting idea they proposed was to choose two bad survival and two good survival lncRNAs and correlate their expression with the clinical outcome of patients. The four lncRNAs chosen were LINC00839 and FIRRE, correlated with bad prognosis and tumor progression, and LOC283177 and LOC101928100, associated with spontaneous regression and neuronal differentiation. The whole of these lncRNAs expression was used to develop a four-lncRNAs signature risk score to predict survival in NB patients [99].

Long intergenic non-protein coding RNA 839 (LINC00839) was also studied in breast cancer [100] and osteosarcoma [101]. Its upregulation correlated with bad prognosis in both cases. In particular, Chen et al. [100] described the mechanism by which this lncRNA acts in breast cancer cells: it is transcriptionally activated by MYC interaction with its promoter and can bind Lin28b, promoting positive regulation of both MYC and Lin28b protein. Despite this mechanism occurring in breast cancer cells, it is interesting to note that *LIN28B* overexpression promotes NB onset [102].

Functional intergenic repeating RNA element (FIRRE) lncRNA was correlated with poor overall survival also in diffuse large B-cell lymphoma (DLBCL). Shi et al. [103] suggested that, in DLBCL, MYC binds *FIRRE* promoter, activating its transcription. In turn, FIRRE activates the WNT/β-catenin pathway, leading to an increase in cell proliferation and reduction of cell apoptosis [103]. Moreover, *FIRRE* upregulation was associated with shorter overall survival in kidney renal clear cell carcinoma, kidney renal papillary cell carcinoma, pancreatic adenocarcinoma, brain low-grade glioma, HCC and mesothelioma [104].

LOC283177, also known as B3GAT1 Divergent Transcript (B3GAT1-DT), is a functionally uncharacterized lncRNA. Its deregulation is reported in DLBCL [105]. Indeed, Conde et al. proposed that this lncRNA could represent a potential susceptibility locus for DLBCL, showing a higher association with this tumor in young patients rather than in the whole cohort [106]. However, further studies are necessary to understand in which way it could be involved in tumorigenesis.

LOC101928100 is also known as KLRK1-Antisense RNA 1 (KLRL1-AS1), but other information about its activity and correlation with cancer is not available. Only Shi et al. mentioned this lncRNA associated with tumor, where they observed a KLRK1-AS1 upregulation in NSCLC compared with non-tumor samples without investigating further [107].

## 5. Conclusions

As we previously reported, NB is characterized by clinical heterogeneity: patients may experience spontaneous regression or incur aggressive tumors with poor patient survival [108]. At the moment, the genetic and molecular mechanisms leading to NB development are largely unknown. In recent years, some new therapeutic approaches were developed, such as GD2 monoclonal antibodies or B7-H3 IgG antibodies, but these are not applicable in all cases [11,13,15].

In this context, the study of lncRNAs assumes particular importance to bring new concepts in order to improve both prognosis and therapeutic approaches. 

In this mini-review, we summarized the more recent insights into the main lncRNAs involved in NB development and progression (Table 1). Indeed, our aim was to emphasize the most recent studies in this field, underlining not only the involvement of lncRNAs in the malignancy process but also in NB regression to strengthen their potential as an indicator for bad/good prognosis. Although many of these studies focused their attention on the molecular mechanisms by which the lncRNAs may act, further research is necessary to understand the real importance and role of each one of these transcripts, with the final goal to improve patients’ survival.

## Figures and Tables

**Table 1:** Role of lncRNAs in neuroblastoma (NB) and in other tumors.

LncRNA	Role in NB	Role in Other Tumors
FOXD3-AS1	Reduced expression	Reduced expression led to induction of *p53*, *c-Myc,* and *retinoblastoma*
	Positive prognostic marker for positive outcome	Reduced expression in colon cancer
	Increased invasion and proliferation rates	
NBAT1	Independent prognostic marker for clinical outcome	Involved in clear cell renal cell carcinoma, ovarian cancer and breast cancer
	Lower expression in HR-NB Tumor suppressor activity through the regulation of *SOX9*, *OSMR* and *VCAN*	Emerging role in pathobiology of glioblastoma
CASC15	Low expression directly correlated with poor diagnosis Neuronal differentiation	High expression in other tumors, including CC, breast cancer, HCC, gastric cancer and melanoma Down-regulated in ovarian cancer and glioma
DLX-AS1	Oncogenic role	Involvement in liver cancer and lung cancer
	Positive correlation with poor differentiation and poor outcome	Aberrant expression in other tumor tissues and associated with tumor progression
	Improper neuronal differentiation	
NDM29	Neuronal differentiation	ND
LncNB1	Cell proliferation regulation	Moderate expression in melanoma
	Poor prognosis prediction	Absent in other tumors
SNHG1	miR338-3p inhibitor	Oncogene and regulator of Wnt/β-catenin axis in CRC
	Proliferation, migration and invasion regulator	*p53* inhibitor in HCC
	Positively correlated with *MYCN* amplification	miR-101-3p/SOX9/Wnt/β-catenin axis regulation in NSCLC
SNHG16	Associated with poor prognosis Regulates cell cycle progression, cell proliferation, migration and autophagy	Correlated with poor prognosis in different types of cancer, including bladder cancer, breast cancer, glioma, HCC, and osteosarcoma
LINC00839	Correlated with bad prognosis and tumor progression	Involved in breast cancer and osteosarcoma
FIRRE	Correlated with bad prognosis and tumor progression	Correlated with poor survival in diffuse large B-cell lymphoma, kidney renal clear cell carcinoma, kidney renal papillary cell carcinoma, pancreatic adenocarcinoma, glioma, HCC, and mesothelioma
LOC283177	Associated with spontaneous regression and neuronal differentiation	Involved in diffuse large B-cell lymphoma
LOC101928100	Associated with spontaneous regression and neuronal differentiation	ND

## Data Availability

Not applicable.

## References

[1] Tsubota S., Kadomatsu K. (2018). Origin and initiation mechanisms of neuroblastoma. Cell Tissue Res..

[2] Brodeur G.M. (2003). Neuroblastoma: Biological insights into a clinical enigma. Nat. Rev. Cancer.

[3] Trigg R.M., Turner S.D. (2018). ALK in neuroblastoma: Biological and therapeutic implications. Cancers.

[4] Monclair T., Brodeur G.M., Ambros P.F., Brisse H.J., Cecchetto G., Holmes K., Kaneko M., London W.B., Matthay K.K., Nuchtern J.G. (2009). The International Neuroblastoma Risk Group (INRG) staging system: An INRG Task Force report. J. Clin. Oncol..

[5] Cohn S.L., Pearson A.D.J., London W.B., Monclair T., Ambros P.F., Brodeur G.M., Faldum A., Hero B., Iehara T., Machin D. (2009). The International Neuroblastoma Risk Group (INRG) classification system: An INRG task force report. J. Clin. Oncol..

[6] Ackermann S., Cartolano M., Hero B., Welte A., Kahlert Y., Roderwieser A., Bartenhagen C., Walter E., Gecht J., Kerschke L. (2018). A mechanistic classification of clinical phenotypes in neuroblastoma. Science.

[7] Maris J.M., Hogarty M.D., Bagatell R., Cohn S.L. (2007). Neuroblastoma. Lancet.

[8] Prajapati B., Fatma M., Fatima M., Khan M.T., Sinha S., Seth P.K. (2019). Identification of lncRNAs Associated With Neuroblastoma in Cross-Sectional Databases: Potential Biomarkers. Front. Mol. Neurosci..

[9] Huang M., Weiss W.A. (2013). Neuroblastoma and MYCN. Cold Spring Harb. Perspect. Med..

[10] Dobrenkov K., Ostrovnaya I., Gu J., Cheung I.Y., Cheung N.K.V. (2016). Oncotargets GD2 and GD3 are highly expressed in sarcomas of children, adolescents, and young adults. Pediatr. Blood Cancer.

[11] Sait S., Modak S. (2017). Anti-GD2 immunotherapy for neuroblastoma. Expert Rev. Anticancer Ther..

[12] Schumacher-Kuckelkorn R., Volland R., Gradehandt A., Hero B., Simon T., Berthold F. (2017). Lack of immunocytological GD2 expression on neuroblastoma cells in bone marrow at diagnosis, during treatment, and at recurrence. Pediatr. Blood Cancer.

[13] Dondero A., Morini M., Cangelosi D., Mazzocco K., Serra M., Spaggiari G.M., Rotta G., Tondo A., Locatelli F., Castellano A. (2021). Multiparametric flow cytometry highlights B7-H3 as a novel diagnostic/therapeutic target in GD2neg/low neuroblastoma variants. J. Immunother. Cancer.

[14] Castriconi R., Dondero A., Augugliaro R., Cantoni C., Carnemolla B., Sementa A.R., Negri F., Conte R., Corrias M.V., Moretta L. (2004). Identification of 4Ig-B7-H3 as a neuroblastoma-associated molecule that exerts a protective role from an NK cell-mediated lysis. Proc. Natl. Acad. Sci. USA.

[15] Modak S., Kramer K., Gultekin S.H., Guo H.F., Cheung N.K.V. (2001). Monoclonal antibody 8H9 targets a novel cell surface antigen expressed by a wide spectrum of human solid tumors. Cancer Res..

[16] Langbein T., Weber W.A., Eiber M. (2019). Future of Theranostics: An Outlook on Precision Oncology in Nuclear Medicine. J. Nucl. Med..

[17] Irwin M.S., Park J.R. (2015). Neuroblastoma: Paradigm for precision medicine. Pediatr. Clin. N. Am..

[18] Mattick J.S., Makunin I.V. (2006). Non-coding RNA. Hum. Mol. Genet..

[19] Louro R., Smirnova A.S., Verjovski-Almeida S. (2009). Long intronic noncoding RNA transcription: Expression noise or expression choice?. Genomics.

[20] Ma L., Bajic V.B., Zhang Z. (2013). On the classification of long non-coding RNAs. RNA Biol..

[21] Natoli G., Andrau J.C. (2012). Noncoding transcription at enhancers: General principles and functional models. Annu. Rev. Genet..

[22] Zhang P., Wu W., Chen Q., Chen M. (2019). Non-Coding RNAs and their Integrated Networks. J. Integr. Bioinform..

[23] Fang Y., Fullwood M.J. (2016). Roles, Functions, and Mechanisms of Long Non-coding RNAs in Cancer. Genom. Proteom. Bioinform..

[24] Pink R.C., Wicks K., Caley D.P., Punch E.K., Jacobs L., Raul D., Carter F. (2011). Pseudogenes: Pseudo-functional or key regulators in health and disease?. RNA.

[25] Tutar Y. (2012). Pseudogenes. Comp. Funct. Genom..

[26] Tsagakis I., Douka K., Birds I., Aspden J.L. (2020). Long non-coding RNAs in development and disease: Conservation to mechanisms. J. Pathol..

[27] Mercer T.R., Dinger M.E., Mattick J.S. (2009). Long non-coding RNAs: Insights into functions. Nat. Rev. Genet..

[28] Bhan A., Mandal S.S. (2014). Long noncoding RNAs: Emerging stars in gene regulation, epigenetics and human disease. ChemMedChem.

[29] Ponting C.P., Oliver P.L., Reik W. (2009). Evolution and Functions of Long Noncoding RNAs. Cell.

[30] Schmitt A.M., Chang H.Y. (2016). Long Noncoding RNAs in Cancer Pathways. Cancer Cell.

[31] Chi Y., Wang D., Wang J., Yu W., Yang J. (2019). Long Non-Coding RNA in the Pathogenesis of Cancers. Cells.

[32] Beckedorff F.C., Sena Amaral M., Deocesano-Pereira C., Verjovski-Almeida S. (2013). Long non-coding RNAs and their implications in cancer epigenetics. Biosci. Rep..

[33] Buechner J., Einvik C. (2012). N-myc and noncoding RNAs in neuroblastoma. Mol. Cancer Res..

[34] Zhao X., Li D., Huang D., Song H., Mei H., Fang E., Wang X., Yang F., Zheng L., Huang K. (2018). Risk-Associated Long Noncoding RNA FOXD3-AS1 Inhibits Neuroblastoma Progression by Repressing PARP1-Mediated Activation of CTCF. Mol. Ther..

[35] Guan Y., Bhandari A., Xia E., Yang F., Xiang J., Wang O. (2019). lncRNA FOXD3-AS1 is associated with clinical progression and regulates cell migration and invasion in breast cancer. Cell Biochem. Funct..

[36] Ma W., Shi S., Chen L., Lou G., Feng X. (2021). SP1-induced lncRNA FOXD3-AS1 contributes to tumorigenesis of cervical cancer by modulating the miR-296-5p/HMGA1 pathway. J. Cell. Biochem..

[37] Yang X., Du H., Bian W., Li Q., Sun H. (2021). FOXD3-AS1/miR-128-3p/LIMK1 axis regulates cervical cancer progression. Oncol. Rep..

[38] Wang L. (2021). ELF1-activated FOXD3-AS1 promotes the migration, invasion and EMT of osteosarcoma cells via sponging miR-296-5p to upregulate ZCCHC3. J. Bone Oncol..

[39] Hu J., Pan J., Luo Z., Duan Q., Wang D. (2020). Long non-coding RNA FOXD3-AS1 silencing exerts tumor suppressive effects in nasopharyngeal carcinoma by downregulating FOXD3 expression via microRNA-185-3p upregulation. Cancer Gene Ther..

[40] Tonini G.P. (2017). The Origin of Neuroblastoma. Neuroblastoma—Current State and Recent Updates.

[41] Lee Y.H., Kim J.H., Song G.G. (2014). Genome-wide pathway analysis in neuroblastoma. Tumor Biol..

[42] Bae J.S., Lee J.W., Yoo J.E., Joung J.G., Yoo K.H., Koo H.H., Song Y.M., Sung K.W. (2020). Genome-wide association study for the identification of novel genetic variants associated with the risk of neuroblastoma in Korean children. Cancer Res. Treat..

[43] Barr E., Applebaum M. (2018). Genetic Predisposition to Neuroblastoma. Children.

[44] Decock A., Ongenaert M., Vandesompele J., Speleman F. (2011). Neuroblastoma epigenetics: From candidate gene approaches to genome-wide screenings. Epigenetics.

[45] Maris J.M., Mosse Y.P., Bradfield J.P., Hou C., Monni S., Scott R.H., Asgharzadeh S., Attiyeh E.F., Diskin S.J., Laudenslager M. (2008). Chromosome 6p22 Locus Associated with Clinically Aggressive Neuroblastoma. N. Engl. J. Med..

[46] Mondal T., Juvvuna P.K., Kirkeby A., Mitra S., Kosalai S.T., Traxler L., Hertwig F., Wernig-Zorc S., Miranda C., Deland L. (2018). Sense-Antisense lncRNA Pair Encoded by Locus 6p22.3 Determines Neuroblastoma Susceptibility via the USP36-CHD7-SOX9 Regulatory Axis. Cancer Cell.

[47] Pandey G.K., Mitra S., Subhash S., Hertwig F., Kanduri M., Mishra K., Fransson S., Ganeshram A., Mondal T., Bandaru S. (2014). The Risk-Associated Long Noncoding RNA NBAT-1 Controls Neuroblastoma Progression by Regulating Cell Proliferation and Neuronal Differentiation. Cancer Cell.

[48] Wang D.L., Yuan P., Tian J.Y. (2021). Expression of long noncoding RNA NBAT1 is associated with the outcome of patients with non-small cell lung cancer. Rev. Assoc. Med. Bras..

[49] Zhao B., Cao P., Hu S., Li F., Kong K., Zu Y. (2019). LncRNA-NBAT-1 modulates esophageal cancer proliferation via PKM2. Am. J. Transl. Res..

[50] Wei L., Ling M., Yang S., Xie Y., Liu C., Yi W. (2021). Long noncoding RNA NBAT1 suppresses hepatocellular carcinoma progression via competitively associating with IGF2BP1 and decreasing c-Myc expression. Hum. Cell.

[51] Xue S., Wang S., Li J., Guan H., Jiang S., Guo Y., Li Q. (2019). LncRNA NBAT1 suppresses cell proliferation and migration via miR-346/GSK-3β axis in renal carcinoma. IUBMB Life.

[52] Xue S., Li Q.W., Che J.P., Guo Y., Yang F.Q., Zheng J.H. (2015). Decreased expression of long non-coding RNA NBAT-1 is associated with poor prognosis in patients with clear cell renal cell carcinoma. Int. J. Clin. Exp. Pathol..

[53] Lei T., Lv Z.Y., Fu J.F., Wang Z., Fan Z., Wang Y. (2018). LncRNA NBAT-1 is down-regulated in lung cancer and influences cell proliferation, apoptosis and cell cycle. Eur. Rev. Med. Pharmacol. Sci..

[54] Hu P., Chu J., Wu Y., Sun L., Lv X., Zhu Y., Li J., Guo Q., Gong C., Liu B. (2015). NBAT1 suppresses breast cancer metastasis by regulating DKK1 via PRC2. Oncotarget.

[55] Pandey G.K., Kanduri C. (2015). Fighting Neuroblastomas with NBAT1. Oncoscience.

[56] Mitra S., Muralidharan S.V., Di Marco M., Juvvuna P.K., Kosalai S.T., Reischl S., Jachimowicz D., Subhash S., Raimondi I., Kurian L. (2021). Subcellular Distribution of p53 by the p53-Responsive lncRNA NBAT1 Determines Chemotherapeutic Response in Neuroblastoma. Cancer Res..

[57] Chen P., Chen R., Guo H., Cheng J., Zhang R., Liu B., Pang J., Cao W. (2021). CASC15 Polymorphisms are Correlated With Breast Cancer Susceptibility in Chinese Han Women. Clin. Breast Cancer.

[58] Yao X.M., Tang J.H., Zhu H., Jing Y. (2021). High expression of LncRNA CASC15 is a risk factor for gastric cancer prognosis and promote the proliferation of gastric cancer. Eur. Rev. Med. Pharmacol. Sci..

[59] Fernando T.R., Contreras J.R., Zampini M., Rodriguez-Malave N.I., Alberti M.O., Anguiano J., Tran T.M., Palanichamy J.K., Gajeton J., Ung N.M. (2017). The lncRNA CASC15 regulates SOX4 expression in RUNX1-rearranged acute leukemia. Mol. Cancer.

[60] Sheng L., Wei R. (2020). Long Non-Coding RNA-CASC15 Promotes Cell Proliferation, Migration, and Invasion by Activating Wnt/β-Catenin Signaling Pathway in Melanoma. Pathobiology.

[61] Shi Y., Gao S., Zheng Y., Yao M., Ruan F. (2019). Lncrna casc15 functions as an unfavorable predictor of ovarian cancer prognosis and inhibits tumor progression through regulation of mir-221/arid1a axis. Onco. Targets Ther..

[62] Wang B., Xu W., Cai Y., Guo C., Zhou G., Yuan C. (2021). CASC15: A Tumor-Associated Long Non-Coding RNA. Curr. Pharm. Des..

[63] Zhang H.-Y., Xing M.-Q., Guo J., Zhao J.-C., Chen X., Jiang Z., Zhang H., Dong Q. (2019). Long noncoding RNA DLX6-AS1 promotes neuroblastoma progression by regulating miR-107/BDNF pathway. Cancer Cell Int..

[64] Li C., Wang S., Yang C. (2020). Long non-coding RNA DLX6-AS1 regulates neuroblastoma progression by targeting YAP1 via miR-497-5p. Life Sci..

[65] Li J., Li P., Zhao W., Yang R., Chen S., Bai Y., Dun S., Chen X., Du Y., Wang Y. (2015). Expression of long non-coding RNA DLX6-AS1 in lung adenocarcinoma. Cancer Cell Int..

[66] Fu X., Tian Y., Kuang W., Wen S., Guo W. (2019). Long non-coding RNA DLX6-AS1 silencing inhibits malignant phenotypes of gastric cancer cells. Exp. Ther. Med..

[67] Zhang J.J., Xu W.R., Chen B., Wang Y.Y., Yang N., Wang L.J., Zhang Y.L. (2019). The up-regulated lncRNA DLX6-AS1 in colorectal cancer promotes cell proliferation, invasion and migration via modulating PI3K/AKT/mTOR pathway. Eur. Rev. Med. Pharmacol. Sci..

[68] Zhao P., Guan H., Dai Z., Ma Y., Zhao Y., Liu D. (2019). Long noncoding RNA DLX6-AS1 promotes breast cancer progression via miR-505-3p/RUNX2 axis. Eur. J. Pharmacol..

[69] Wang H., Niu X., Jiang H., Mao F., Zhong B., Jiang X., Fu G. (2020). Long non-coding RNA DLX6-AS1 facilitates bladder cancer progression through modulating miR-195-5p/VEGFA signaling pathway. Aging.

[70] Kong L., Zhang C. (2020). LncRNA DLX6-AS1 aggravates the development of ovarian cancer via modulating FHL2 by sponging miR-195-5p. Cancer Cell Int..

[71] Zhang N., Meng X., Mei L., Zhao C., Chen W. (2019). LncRNA DLX6-AS1 promotes tumor proliferation and metastasis in osteosarcoma through modulating miR-641/HOXA9 signaling pathway. J. Cell. Biochem..

[72] Pagano A., Castelnuovo M., Tortelli F., Ferrari R., Dieci G., Cancedda R. (2007). New small nuclear RNA gene-like transcriptional units as sources of regulatory transcripts. PLoS Genet..

[73] Castelnuovo M., Massone S., Tasso R., Fiorino G., Gatti M., Robello M., Gatta E., Berger A., Strub K., Florio T. (2010). An Alu-like RNA promotes cell differentiation and reduces malignancy of human neuroblastoma cells. FASEB J..

[74] Alloisio S., Garbati P., Viti F., Dante S., Barbieri R., Arnaldi G., Petrelli A., Gigoni A., Giannoni P., Quarto R. (2017). Generation of a Functional Human Neural Network by NDM29 Overexpression in Neuroblastoma Cancer Cells. Mol. Neurobiol..

[75] Vella S., Penna I., Longo L., Pioggia G., Garbati P., Florio T., Rossi F., Pagano A. (2015). Perhexiline maleate enhances antitumor efficacy of cisplatin in neuroblastoma by inducing over-expression of NDM29 ncRNA. Sci. Rep..

[76] Costa D., Gigoni A., Würth R., Cancedda R., Florio T., Pagano A. (2014). Metformin inhibition of neuroblastoma cell proliferation is differently modulated by cell differentiation induced by retinoic acid or overexpression of NDM29 non-coding RNA. Cancer Cell Int..

[77] Gavazzo P., Vella S., Marchetti C., Nizzari M., Cancedda R., Pagano A. (2011). Acquisition of neuron-like electrophysiological properties in neuroblastoma cells by controlled expression of NDM29 ncRNA. J. Neurochem..

[78] Garbati P., Barbieri R., Cangelosi D., Zanon C., Costa D., Eva A., Thellung S., Calderoni M., Baldini F., Tonini G.P. (2020). Mcm2 and carbonic anhydrase 9 are novel potential targets for neuroblastoma pharmacological treatment. Biomedicines.

[79] Liu P.Y., Tee A.E., Milazzo G., Hannan K.M., Maag J., Mondal S., Atmadibrata B., Bartonicek N., Peng H., Ho N. (2019). The long noncoding RNA lncNB1 promotes tumorigenesis by interacting with ribosomal protein RPL35. Nat. Commun..

[80] Thin K.Z., Tu J.C., Raveendran S. (2019). Long non-coding SNHG1 in cancer. Clin. Chim. Acta.

[81] Yang H., Wang S., Kang Y.J., Wang C., Xu Y., Zhang Y., Jiang Z. (2018). Long non-coding RNA SNHG1 predicts a poor prognosis and promotes colon cancer tumorigenesis. Oncol. Rep..

[82] Zhang M., Wang W., Li T., Yu X., Zhu Y., Ding F., Li D., Yang T. (2016). Long noncoding RNA SNHG1 predicts a poor prognosis and promotes hepatocellular carcinoma tumorigenesis. Biomed. Pharm..

[83] Cui Y., Zhang F., Zhu C., Geng L., Tian T., Liu H. (2017). Upregulated lncRNA SNHG1 contributes to progression of nonsmall cell lung cancer through inhibition of miR-101-3p and activation of Wnt/β-catenin signaling pathway. Oncotarget.

[84] Chen Y., Lian Y., Ma Y., Wu C., Zheng Y., Xie N. (2018). LncRNA SNHG1 promotes α-synuclein aggregation and toxicity by targeting miR-15b-5p to activate SIAH1 in human neuroblastoma SH-SY5Y cells. Neurotoxicology.

[85] Zhang N., Liu F.L., Ma T.S., Zeng Z.D., Zhang J.J. (2019). LncRNA SNHG1 contributes to tumorigenesis and mechanism by targeting MIR-338-3p to regulate PLK4 in human neuroblastoma. Eur. Rev. Med. Pharmacol. Sci..

[86] Sahu D., Hsu C.L., Lin C.C., Yang T.W., Hsu W.M., Ho S.Y., Juan H.F., Huang H.C. (2016). Co-expression analysis identifies long noncoding RNA SNHG1 as a novel predictor for event-free survival in neuroblastoma. Oncotarget.

[87] Gong C.Y., Tang R., Nan W., Zhou K.S., Zhang H.H. (2020). Role of SNHG16 in human cancer. Clin. Chim. Acta.

[88] Feng F., Chen A., Huang J., Xia Q., Chen Y., Jin X. (2018). Long noncoding RNA SNHG16 contributes to the development of bladder cancer via regulating miR-98/STAT3/Wnt/β-catenin pathway axis. J. Cell. Biochem..

[89] Cai C., Huo Q., Wang X., Chen B., Yang Q. (2017). SNHG16 contributes to breast cancer cell migration by competitively binding miR-98 with E2F5. Biochem. Biophys. Res. Commun..

[90] Zhou X.Y., Liu H., Ding Z., Xi H.P., Wang G.W. (2020). lncRNA SNHG16 promotes glioma tumorigenicity through miR-373/EGFR axis by activating PI3K/AKT pathway. Genomics.

[91] Xie X., Xu X., Sun C., Yu Z. (2019). Long intergenic noncoding RNA SNHG16 interacts with miR-195 to promote proliferation, invasion and tumorigenesis in hepatocellular carcinoma. Exp. Cell Res..

[92] Su P., Mu S., Wang Z. (2019). Long Noncoding RNA SNHG16 Promotes Osteosarcoma Cells Migration and Invasion via Sponging miRNA-340. DNA Cell Biol..

[93] Liu S., Zhang W., Liu K., Liu Y. (2019). LncRNA SNHG16 promotes tumor growth of pancreatic cancer by targeting miR-218-5p. Biomed. Pharm..

[94] Tao L., Wang X., Zhou Q. (2020). Long noncoding RNA SNHG16 promotes the tumorigenicity of cervical cancer cells by recruiting transcriptional factor SPI1 to upregulate PARP9. Cell Biol. Int..

[95] Yu Y., Chen F., Yang Y., Jin Y., Shi J., Han S., Chu P., Lu J., Tai J., Wang S. (2019). LncRNA SNHG16 is associated with proliferation and poor prognosis of pediatric neuroblastoma. Int. J. Oncol..

[96] Xu Z., Sun Y., Wang D., Sun H., Liu X. (2020). SNHG16 promotes tumorigenesis and cisplatin resistance by regulating miR-338-3p/PLK4 pathway in neuroblastoma cells. Cancer Cell Int..

[97] Bao J., Zhang S., Meng Q., Qin T. (2020). SNHG16 Silencing Inhibits Neuroblastoma Progression by Downregulating HOXA7 via Sponging miR-128-3p. Neurochem. Res..

[98] Wen Y., Gong X., Dong Y., Tang C. (2020). Long non coding RNA SNHG16 facilitates proliferation, migration, invasion and autophagy of neuroblastoma cells via sponging miR-542-3p and upregulating ATG5 expression. Onco. Targets Ther..

[99] Meng X., Fang E., Zhao X., Feng J. (2020). Identification of prognostic long noncoding RNAs associated with spontaneous regression of neuroblastoma. Cancer Med..

[100] Chen Q., Shen H., Zhu X., Liu Y., Yang H., Chen H., Xiong S., Chi H., Xu W. (2020). A nuclear lncRNA Linc00839 as a Myc target to promote breast cancer chemoresistance via PI3K/AKT signaling pathway. Cancer Sci..

[101] Zhang Y., Guo H., Ma L., Chen X., Chen G. (2020). Long noncoding rna linc00839 promotes the malignant progression of osteosarcoma by competitively binding to microrna-454-3p and consequently increasing c-met expression. Cancer Manag. Res..

[102] Corallo D., Donadon M., Pantile M., Sidarovich V., Cocchi S., Ori M., De Sarlo M., Candiani S., Frasson C., Distel M. (2020). LIN28B increases neural crest cell migration and leads to transformation of trunk sympathoadrenal precursors. Cell Death Differ..

[103] Shi X., Cui Z., Liu X., Wu S., Wu Y., Fang F., Zhao H. (2019). Biochemical and Biophysical Research Communications LncRNA FIRRE is activated by MYC and promotes the development of diffuse large B-cell lymphoma via Wnt/b -catenin signaling pathway. Biochem. Biophys. Res. Commun..

[104] Li Y., Huang S., Wei Z., Yang B.O. (2020). A putative competing endogenous RNA network in cisplatin—resistant lung adenocarcinoma cells identifying potentially rewarding research targets. Oncol. Lett..

[105] Nobili L., Ronchetti D., Taiana E., Neri A. (2017). Long non-coding RNAs in B-cell malignancies: A comprehensive overview. Oncotarget.

[106] Conde L., Riby J., Zhang J., Bracci P.M., Skibola C.F. (2014). Copy number variation analysis on a non-hodgkin lymphoma case-control study identifies an 11q25 duplication associated with diffuse large B-cell lymphoma. PLoS ONE.

[107] Shi X., Ma C., Zhu Q., Yuan D., Sun M., Gu X., Wu G., Lv T., Song Y. (2016). Upregulation of long intergenic noncoding RNA 00673 promotes tumor proliferation via LSD1 interaction and repression of NCALD in non-small-cell lung cancer. Oncotarget.

[108] Johnsen J.I., Dyberg C., Wickström M. (2019). Neuroblastoma—A neural crest derived embryonal malignancy. Front. Mol. Neurosci..

